# Community-based trial assessing the impact of annual versus semiannual mass drug administration with ivermectin plus albendazole and praziquantel on helminth infections in northwestern Liberia

**DOI:** 10.1016/j.actatropica.2022.106437

**Published:** 2022-07

**Authors:** Obiora A. Eneanya, Lincoln Gankpala, Charles W. Goss, Aaron T. Momolu, Enoch S. Nyan, Emmanuel B. Gray, Kerstin Fischer, Kurt Curtis, Fatorma K. Bolay, Gary J. Weil, Peter U. Fischer

**Affiliations:** a Department of Medicine, Infectious Diseases Division, Washington University School of Medicine, St. Louis, MO, United States; b Division of Public Health and Medical Research, National Public Health Institute of Liberia, Charlesville, Liberia; c Division of Biostatistics, Washington University School of Medicine, St. Louis, MO, United States; d Ministry of Health of Liberia, C.B. Dunbar Hospital, Gbarnga, Liberia

**Keywords:** Lymphatic filariasis, Onchocerciasis, Soil-transmitted helminths, Schistosoma mansoni, Mass drug administration, Liberia

## Abstract

We assessed the impact of three annual vs five semiannual rounds of mass drug administration (MDA) with ivermectin plus albendazole followed by praziquantel for the control or elimination of lymphatic filariasis (LF), onchocerciasis, soil-transmitted helminth (STH) infections and schistosomiasis in Lofa County, Liberia. The study started in 2012 and was interrupted in 2014 during the Ebola virus outbreak. Repeated cross-sectional surveys were conducted in individuals 5 years and older to measure infection markers. *Wuchereria bancrofti* antigenemia prevalences decreased from 12.5 to 1.2% (90% reduction) and from 13.6 to 4.2% (69% reduction) one year after three rounds of annual or five rounds of semiannual MDA, respectively. Mixed effects logistic regression models showed decreases in odds of antigenemia positivity were 91 and 74% at that time in the annual and semiannual treatment zones, respectively (*p* < 0.001). Semiannual MDA was slightly more effective for reducing *Onchocerca volvulus* microfiladermia prevalence and at follow-up 3 were 74% (from 14.4 to 3.7%) and 83% (from 23.6 to 4.5%) in the annual and semiannual treatment zones, respectively. Both treatment schedules had similar beneficial effects on hookworm prevalence. Thus, annual and semiannual MDA with ivermectin and albendazole had similar beneficial impacts on LF, onchocerciasis, and STH in this setting. In contrast, MDA with praziquantel had little impact on hyperendemic *Schistosoma mansoni* in the study area. Results from a long-term follow-up survey showed that improvements in infection parameters were sustained by routine annual MDA provided by the Liberian Ministry of Health after our study endpoint.

## Highlights

• Mass drug administration (MDA) with ivermectin plus albendazole was more effective for clearing bancroftian filariasis than it was for onchocerciasis or hookworm.
• Semiannual MDA was no more effective than annual MDA for reducing prevalences of *W. bancrofti, O. volvulus*, or hookworm infections.
• Decreases in bancroftian filariasis prevalence were achieved despite unavoidable interruptions in our study related to the 2014 West Africa Ebola epidemic and the 2020 SARS-CoV-2 pandemic.
• Neglected Tropical Disease elimination programs in areas coendemic for lymphatic filariasis and onchocerciasis should focus on delivering high quality annual MDA with high coverage and compliance and not try to stretch limited resources to deliver semiannual MDA.
• MDA with praziquantel had little impact on *Schistosoma mansoni* infection in the heavily infected area.

## 1 Introduction

Liberia is endemic for many Neglected Tropical Diseases (NTDs) (Republic of Liberia Ministry of Health, 2015). These diseases often cause significant morbidity and disproportionately affect poorer populations living in rural areas where inadequate housing and sanitation are common. Liberia is a post-conflict country that endured nearly 14 years of civil war (1990–2003), during which time NTD control programs were suspended in most parts of the country. Following the end of the civil war, the Liberian national NTD program prioritized lymphatic filariasis (LF, caused by *Wuchereria bancrofti*), onchocerciasis, schistosomiasis, and soil-transmitted helminth (STH) infections for control and/or elimination. Nationwide mapping of these major NTDs began in 2010, and large scale preventive chemotherapy started in some areas in 2012 (Republic of Liberia Ministry of Health, 2015). Population-based mass-drug administration (MDA) using ivermectin and albendazole is used for LF and onchocerciasis control. Albendazole and praziquantel was provided to school-aged children for schistosomiasis and STH control.

In March 2014, the first case of Ebola virus disease in Liberia was reported in Foya town in Lofa County, near Liberia's border with Guinea and Sierra Leone (Kouadio et al., 2015). Subsequently Lofa County became an early epicenter of the Ebola outbreak in Liberia; 451 confirmed or probable deaths due to Ebola occurred in the area during the outbreak. The country was declared Ebola free in March 2015 (Thomas et al., 2017). The response to the outbreak drained resources away from other health programs. For example, MDA rounds for NTD control were missed and delivery of bed nets for mosquito control and malaria prevention was interrupted during this period. Health workers who were normally employed as community drug distributors were redeployed to focus on efforts aimed at bringing the outbreak under control (Thomas et al., 2017; Hotez, 2015; Gray et al., 2021). At the end of the outbreak, when it was safe to return to the field, our team conducted a survey in Lofa County to assess community attitude towards restarting MDA and the general preparedness for resuming public health programs in this area (Bogus et al., 2016).

This study was one of several parallel studies that were conducted as part of the DOLF project (www.dolfproject.wustl.edu) to compare the impact of annual and semiannual MDA on helminthic infections (Supali et al., 2019; Eneanya et al., 2021). A computer modeling study predicted that semiannual MDA should accelerate LF elimination (Stolk et al., 2013). However, simulation modeling relies on assumptions for parameters such as endemicity level, treatment coverage, compliance rates, treatment efficacy, and other biological parameters that can influence transmission and clearance of infections. Population-based field studies are required to test model predictions.

In this study, we performed repeated cross-sectional community parasitology surveys to compare the impact of three rounds of annual vs five rounds of semiannual MDA on LF and other coendemic helminth infections. Two additional annual MDA rounds were provided by the Ministry of Health, and this allowed us to assess whether improvements observed at the primary endpoint of the study were sustained by routine annual MDA.

## 2 Materials and methods

### 2.1 Study area

**Fig. 1:**
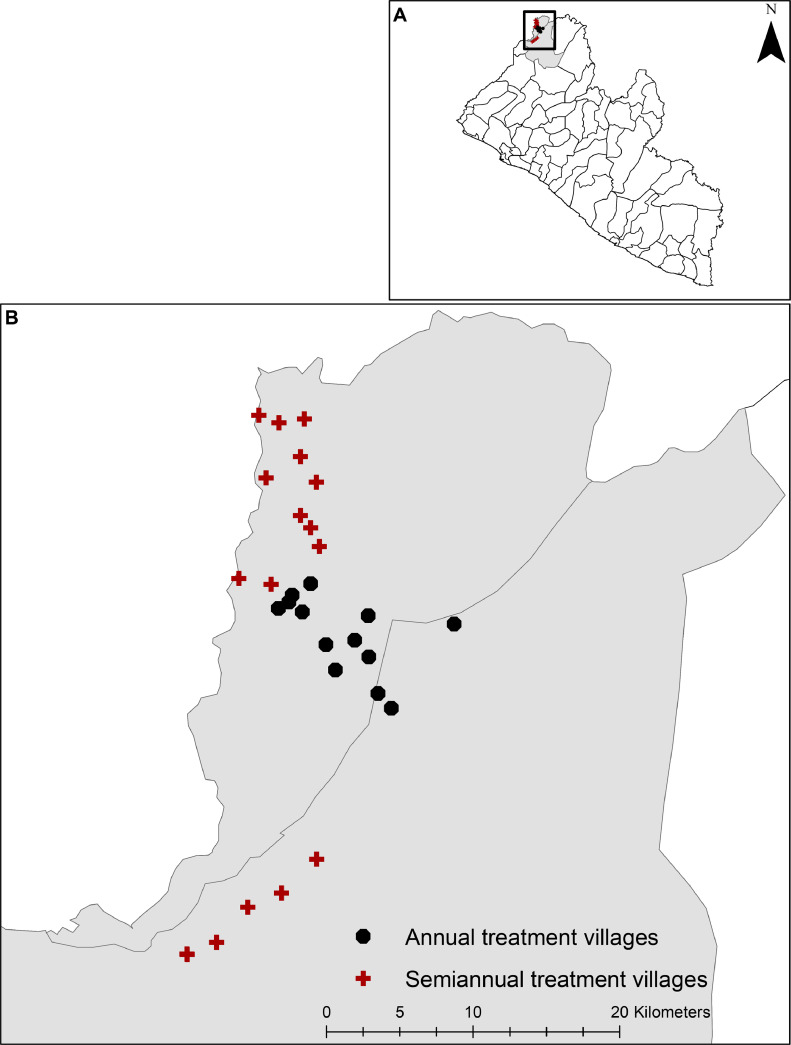
Maps of Liberia (top) and Lofa County (bottom) with study villages shown in the North, Center, and South MDA treatment zones.

The study was conducted in 32 villages in Foya and Kolahun districts within Lofa County, Liberia (Fig. 1). Lofa is situated in the northernmost part of the country and has an approximate population of 370,000, making it the third most populous county in Liberia. It shares borders with Sierra Leone to the west and Guinea to the north and east. The primary agricultural products of Lofa are rice, cassava, cocoa, coffee, and rubber. Previous studies as well as mapping efforts by the Liberian government showed that the county was endemic for both LF and onchocerciasis (Republic of Liberia Ministry of Health 2015; Diller, 1947; Kuhlow and Zielke, 1976). Ivermectin MDA for onchocerciasis was provided in Lofa in 2011, but no prior MDA with ivermectin plus albendazole for LF or praziquantel MDA for schistosomiasis was provided prior to our study.

### 2.2 Parasitological surveys and mass drug administration

Provision of annual or semiannual MDA to study villages was based on results of baseline surveys. The study area was divided into three treatment zones (North, South, and Center, Fig. 1). Villages in the Center had moderately high endemicity for both LF and onchocerciasis, so this area received annual MDA (Table 1). Villages in the North (high LF and low onchocerciasis) received semiannual MDA, and LF results in this area were compared to those in the Center. LF prevalence was low in the South where onchocerciasis was more prevalent; villages in the South also received semiannual. Therefore, results from the Center and South villages were used to compare the effects of annual and semiannual MDA on onchocerciasis. *Schistosoma mansoni* and hookworm prevalences were similar in all three treatment zones.

**Table 1:** Prevalence of helminth infection at baseline stratified by village zone.

		**Lymphatic filariasis**		**Onchocerciasis**		**Soil-transmitted helminths**				**Schistosomiasis**
			***Wuchereria bancrofti***		***Onchocerca volvulus***		***Ascaris lumbricoides***	***Necator americanus***	Trichuris trichiura*	***Schistosoma mansoni***
		**N**	**% (95% CI)**	**N**	**% (95% CI)**	**N**	**% (95% CI)**	**% (95% CI)**	**% (95% CI)**	**% (95% CI)**
**Village locations**	**Center**	996	12.5 (10.6, 14.8)	1008	14.4 (12.3, 16.7)	762	2.2 (1.4, 3.5)	47.5 (44.0, 51.1)	0.5 (0.1, 1.3)	90.7 (88.4, 92.6)
	**North**	1010	13.6 (13.5, 18.1)	1266	5.3 (4.1, 6.7)	1017	0.8 (0.3, 1.5)	43.7 (40.6, 46.8)	1.5 (0.8, 2.4)	89.4 (87.3, 91.2)
	**South**	1146	2.4 (1.6, 3.4)	1142	23.6 (21.2, 26.2)	891	0.6 (0.2, 1.3)	93.2 (91.3, 94.7)	0.7 (0.2, 1.5)	82.9 (80.3, 85.3)

N = Number of participants, CI = Confidence interval, % = Prevalence estimates, *As identified by microscopy only.

For lymphatic filariasis; villages in the Center received annual mass drug administration (MDA) while villages in the North received semiannual MDA. Prevalence estimates were too low in the South clusters and thus these villages was subsequently excluded from the study.

For onchocerciasis; villages in the Center received annual MDA while villages in the South received semiannual MDA. Prevalence estimates were too low in the North clusters and thus these villages was subsequently excluded from the study.

For soil-transmitted helminths and schistosomiasis; villages in the Center received annual MDA while villages in the North and South received semiannual MDA.

**Fig. 2:**
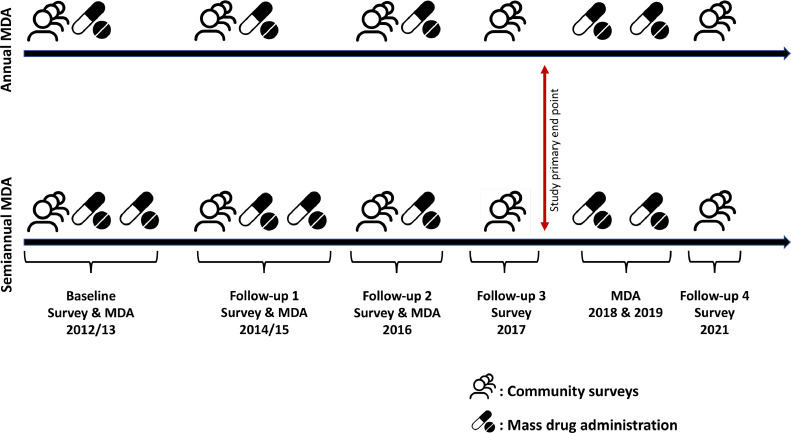
Study timeline indicating time for community parasitological surveys and mass drug administration.

Fig. 2 illustrates the timeline for the study. Five cross-sectional parasitological surveys were conducted between 2012 and 2021. Baseline infection prevalence surveys were conducted in Q3 of 2012. The first and second rounds of MDA were delivered in Q4 2012 and Q2 2013 (the latter for villages that received semiannual MDA only) respectively. For areas that received semiannual treatment, MDA was spaced at 6-month intervals. Follow-up 1 surveys were concluded in Q2 2014; this survey was about two-thirds complete when it had to be suspended due to the Ebola virus disease outbreak in the area. The third round of MDA was delivered to the study communities in Q2 2015 (one year later than planned) after the Ebola outbreak ended. In the villages assigned to semiannual treatment, the fourth round of MDA was delivered in Q4 2015. Follow-up 2 surveys started in Q2 2016, followed by a final round of MDA in both treatment zones in Q3 2016. Follow-up 3 survey, marking the primary endpoint of the study, was conducted in Q2 2017, 12 months after the last MDA round. The study took approximately 18 months longer than initially planned because of the Ebola pause. The study team for this project provided the only MDA in the study area between 2012 and 2017.

Two additional MDA rounds were provided at yearly (Q2 2018 and 2019) intervals by the Liberian Ministry of Health. The SARS-CoV-2 pandemic interrupted plans for a final follow up survey scheduled for Q2 2020. Our team was able to conduct a final survey in Q2 2021 when COVID mitigation procedures were in place. This final (follow-up 4) survey aimed to assess whether the improvements recorded during the formal study period were sustained by MDA provided by the Ministry of Health.

Study personnel provided directly observed MDA to residents of study villages. The MDA regimen included ivermectin (200 µg/kg, dosed using a dosing pole) plus albendazole (a fixed dose of 400 mg). Praziquantel (60 mg/kg, Biltricide, Bayer India) was offered to study participants three days after ivermectin plus albendazole to minimize additive or overlapping adverse events. Community surveys were conducted and households with eligible participants were randomly selected. Individuals ≥ 5 years of age without any evidence of acute illness or severe chronic disease were eligible to participate in the study. Pregnant women were excluded. Treatment compliance was estimated based on surveyed responses obtained during the next parasitological survey, usually one year after the prior round of MDA. Respondents were asked whether they received ivermectin and albendazole in the previous year. These responses where then collated and presented as percentages.

### 2.3 Diagnosis of filarial infections

Lymphatic filariasis: Circulating filarial antigenemia (CFA) was assessed with either the Binax Filariasis Now test (immunochromatographic test (ICT), Alere, Scarborough, Maine, USA) or Filariasis Test Strips (FTS, Alere) (Weil et al., 2013; Weil et al., 1997). CFA testing was performed using fresh capillary day blood according to manufacturer instructions. Both tests (i.e. ICT and FTS) were independently read and scored by two readers. In rare instances of discordant results, a final scoring decision was made by a supervisor.

Night blood was collected from persons with positive CFA results for detection of microfilaria (Mf) in night blood. 250 microliters (µL) of finger prick blood was collected into EDTA tubes between the hours of 9 pm and 11 pm. Mf detection was performed by three line night blood smear. Samples were transported to a field laboratory for further analysis. Thick blood smears were prepared the next morning by placing 60 µL on glass slides in three horizontal lines. Slides were left to dry for 2 days, gently dehemoglobinized with distilled water for 3 min, air dried, fixed with methanol for 1 min, and stained with Giemsa for 15 min. Slides were then examined by microscopy for the presence of Mf.

Onchocerciasis:*O. volvulus* infections were detected by skin snip testing. Here, one skin snip was taken from each posterior iliac crest using a sterile 2 mm Holth-type corneoscleral punch (Everhards GmbH, Meckenheim, Germany). Snips were weighed and then incubated in 100 µL phosphate buffered saline for 24 h in flat-bottomed 96 well microtiter plates at room temperature. Mf that emerged from skin snips were detected by microscopy.

Soil-transmitted helminth infections and intestinal schistosomiasis: Study participants were asked to place a walnut-sized piece of stool in a plastic container. Each container was labelled with unique barcode and the participant's name. Samples were stored in a cooler and transported to the field laboratory. Duplicate Kato-Katz smears were prepared for detection of helminth ova. (WHO 1991) Slides were read within 1 h after preparation by two independent microscopists. A random selection of 10% of the slides were reexamined by a supervisor to ensure quality control. If discrepancies were detected, all slides from that day's collection were reexamined.

### 2.4 Data management and statistical analysis

Tablet computers running an Android operating system were installed with the Epi Info v7 mobile software application (Centers for Disease Control and Prevention, Atlanta, GA). This was used for collecting demographic data in the field. Data were deidentified and encrypted before being transferred to an Azure cloud serve (Microsoft, Redmond, WA). Laboratory results were recorded on paper forms. Barcode identifiers on sample collection tubes matched those used on laboratory result forms. Laboratory results were subsequently transferred with double data entry into Epi Info. Data were downloaded from the server and converted into Microsoft Excel or other software programs for analysis.

Sample size calculations were made using a binomial power calculator based on the following assumptions: alpha = 0.05, two-tailed tests, and power = 0.80. The sample size number per treatment zone was adequate to indicate that filarial antigen rates are less than 2% (a conservative elimination target for LF) with 95% confidence and 80% power, assuming an expected rate of 0.5%. A sample size of at least 335 participants per treatment zone per time point was estimated. These calculations considered various contingencies to assess robustness of the sample size estimates.

STH infection intensities correspond to eggs per gram (epg) of stool. These values were classified as low, moderate or high intensity infections for *Ascaris*, hookworm and *Trichuris* based on World Health Organization (WHO) thresholds (WHO 1991; WHO 1987) Arithmetic mean epg was calculated using data from all participants in the study, whereas geometric mean epg calculations was restricted to only participants with at least one egg count. For onchocerciasis, skin snip Mf counts of <10, 11–30, and > 30 Mf/mg skin were classified as low, moderate, and high intensity infections, respectively.

All cross-sectional surveys were considered to be independent, so unpaired statistical tests were used. The primary outcome of the study was differences in infection prevalences from baseline to follow-up 3. Unadjusted prevalence estimates 95% exact confidence intervals were calculated for each survey. Fisher's exact test was used to assess the statistical significance of changes in infection prevalences between MDA rounds. Mixed effects logistic regression models were used to evaluate the differences in the odds of infection between treatment zones and changes in the odds of infection over time. For this analysis, village was used as the random effects variable to account for potential correlation among subjects within a local area. We assessed the treatment regimen, time, and their interaction. The interaction term was excluded from the final model if it was not significant (*p* < 0.05), otherwise it was retained. Age and gender were included as covariates. Antigenemia prevalence was used as the main LF outcome variable, because microfilaraemia prevalences were too low at baseline and zero for both treatment groups at follow-up 2 and 3 surveys. This was believed to be due to prior distribution of ivermectin MDA for onchocerciasis control by the Ministry of Health in the entire study area.

The geometric mean infection intensity for all helminth infections studies was calculated with data from persons with positive infections. The community microfilarial load (CMFL) was calculated as the geometric mean number of Mf/mL of blood (for LF) and Mf/mg skin snip (for onchocerciasis) using a log(X_1_+1) transformation, where X is the microfilaremia count of all subjects in the study including those without Mf (Remme et al., 1986; Ramzy et al., 2006) All statistical analysis were performed using software packages R (v 4.0.3) (R Development Core Team, 2019) and SAS (v 9.4, SAS Inst. Inc., Cary, NC, USA).

### 2.5 Ethical approval

The study protocol was reviewed and approved by institutional review boards at Washington University School of Medicine (Institutional Review Board ID number: 201107185) and at the University of Liberia (FWA00004982). This study was a registered clinical trial (ClinicalTrials.gov Identified: NCT01905436). Researchers met with national and district level Ministry of Health officials and with leaders from all study village leaders to explain the study goals and procedures prior to the field study. Oral informed consent was obtained for each participant. Enrollment of minors required their assent plus informed consent from at least one parent or guardian.

## 3 Results

### 3.1 Baseline characteristics of the study population and surveyed MDA compliance

Table 2 with data from the baseline survey shows that participants in the two treatment zones were similar with regards to sex ratio, age, bed net use, latrine ownership and screens in their homes. The reported MDA compliance rates in follow-up surveys 1 and 2 were excellent in both treatment zones but higher in the semiannual treatment zone. Reported compliance was lower in follow-up survey 3 (post Ebola) and also lower follow-up survey 4, which followed MDA provided by the Ministry of Health.

**Table 2:** Characteristics of persons enrolled in the baseline survey and surveyed mass drug administration (MDA) compliance.

**Variable**	**Annual treatment zone**	Semiannual treatment zone*
**Persons enrolled in the baseline survey**	1015	2449
**Median age in years (range)**	23 (range 5–97)	21 (range 5–100)
**Gender (Female)**	556 (54.8%)	1312 (53.6%)
**Bed net usage**^1^	523 (51.5%)	1393 (56.9%)
**Door/window screen in house**	9 (0.9%)	111 (4.5%)
**Latrine ownership**	196 (19.3%)	1265 (51.7%)
**Surveyed MDA compliance**^2^		
Baseline	NA	NA
Follow up 1	72.6%	79.1%
Follow up 2	74.0%	82.7%
Follow up 3	64.2%	68.7%
Follow up 4	55.8%	52.5%

See Supplementary 1 for MDA compliance rates stratified by North, Center, and South zones.

1 Bed net usage was defined as persons who slept under bed net the night prior to survey.

2 MDA-compliant participants were those who reported having swallowing albendazole and ivermectin in the previous round of MDA at the time of the next round.

⁎ Includes person's in the North and South zones that received semiannual MDA

### 3.2 Baseline risk factors for *W. bancrofti* infection

**Fig. 3:**
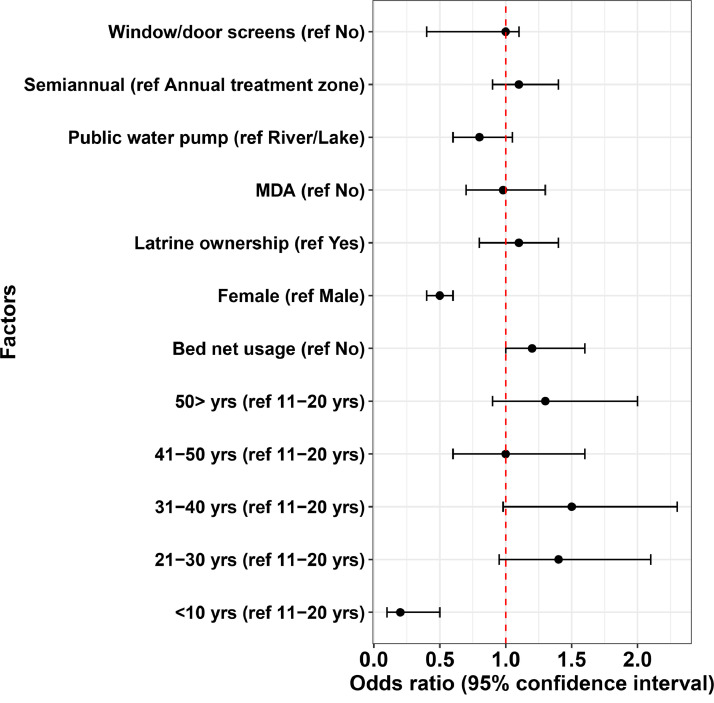
Univariable analysis of the risk factors of filariasis at baseline. The dashed red line indicates an odds ratio of 1.

A univariable analysis was used to identify risk factors for filarial infection at baseline. Here, filarial infection was defined as a positive CFA test (ICT). Fig. 3 shows persons who reported having used bed nets the previous night had higher odds of positivity (odds ratio: 1.2, 95% CI: 1-1.6. *p* = 0.08). Persons in the semiannual MDA zone had similar baseline odds of CFA positivity (odds ratio: 1.1, 95% CI: 0.9-1.4. *p* = 0.47). The odds for LF infection was lower in females than in males (odds ratio: 0.5, 95% CI: 0.4–0.6 *p* < 0.001). Children <10 years of age had significantly lower odds of infection compared to persons 11–20 years of age.

### 3.3 Impact of annual vs semiannual MDA on filarial infection

Table 3 shows that baseline *W. bancrofti* CFA prevalences, geometric mean Mf/ml, and CMFL were similar in the annual and semiannual treatment zones. Baseline Mf prevalences were low in both treatment zones, because they received MDA with ivermectin a few months before to our baseline survey. CFA prevalence decreased by 90% (from 12.5% to 1.2%, *p* = 0.002) after 3 rounds of annual MDA and by 69% (from 13.6 to 4.2%, *p* = 0.026) after 5 rounds of semiannual MDA. ICT and FTS were both used to detect CFA in participants during follow-up 3. Prevalence of antigenemia measured using ICT in the (North) annual treatment zone was 1.3 (95% CI: 0.7, 2.2); and 3.8 (95% CI: 2.8, 5.0) in the (Center) semiannual treatment zone. Similar results were obtained by FTS in the annual and semiannual MDA zones during follow-up 3: 1.2 (95% CI: 0.7, 2.1); and 4.2 (95% CI: 3.2, 5.5). Geometric mean estimates and CMFL calculations were based only on persons with positive Mf counts; too few positive results were obtained for these calculations to be meaningful after the baseline surveys in the annual MDA area and after the first follow-up in the semiannual MDA area.

**Table 3:** Impact of mass drug administration on lymphatic filariasis infection parameters.

**Treatment Zone**	**Timing of the survey**	**Number of participants (N)**	**CFA prevalence(95% CI)**	**Mf prevalence(95% CI)**	**Geometric mean Mf/mL**	**Community Mf load (CMFL)**
						
**Annual MDA**	Baseline^A^	997	12.5 (10.5, 14.8)	1.6 (0.8, 2.5)	59.4 (27.3, 129.2)	3.9 (3.3, 4.7)
	Follow-up 1^A^	639	6.1 (4.4, 8.2)	0 (0.0, 0.6)	N/A	N/A
	Follow-up 2^B^	898	1.8 (1.0, 2.9)	0 (0.0, 0.4)	N/A	N/A
	Follow-up 3^A^^,^^B^	1066	1.2 (0.7, 2.1) B	0 (0.0, 0.3)	N/A	N/A
	Follow-up 4^B^^,^^C^	ND	ND	ND	ND	ND
						
**Semiannual MDA**	Baseline^A^	1169	13.6 (11.7, 15.7)	1.7 (0.7, 2.1)	65.8 (37.4, 116.0)	4.1(3.6, 4.6)
	Follow-up 1^A^	1174	10.1 (8.4, 11.9)	0.2 (0.003, 0.06)	N/A	N/A
	Follow-up 2^B^	1132	3.3 (2.3, 4.5)	0 (0.0, 0.3)	N/A	N/A
	Follow-up 3^A^^,^^B^	1209	4.2 (3.2, 5.5) B	0 (0.0, 0.3)	N/A	N/A
	Follow-up 4^B^^,^^C^	1748	2.6 (2.0, 3.5)	0 (0.0, 0.2)	N/A	N/A

ND, Not done. N/A, Not applicable.

Villages in the Center received annual MDA while villages in the North received semiannual MDA.

A Circulating filarial antigenemia (CFA) was detected by ICT.

B CFA was detected by FTS.

C Both treatment zones received once/year MDA from the Liberia Ministry of Health from months 36 to 60.

We used mixed effects logistic regression models to assess the differences in odds of CFA infection between treatment groups over time. There was a significant (*p* = 0.003) treatment-by-time interaction. The between-treatment odds ratio estimates ranged from 0.98 (95% CI: 0.62, 1.56) (at baseline) to 1.79 (95% CI: 1.05, 3.07) (at follow-up 3), none of the results were significantly different (*p* > 0.10 for all estimates). The decline in CFA prevalence over time was stronger in the annual treatment zone compared to the semiannual treatment zone (Table 4). Relative to baseline, the decline in odds of positivity in the annual treatment zone was 57% at follow-up 1 (*p* < 0.001) and 91% at follow-up 3 (*p* < 0.001). Whereas, in the semiannual treatment zone, the decline in odds of positivity ranged from 22% at follow-up 1 (*p* = 0.257) and 74% at follow-up 3 (*p* < 0.001).

**Table 4:** Adjusted odds ratio and 95% confidence intervals from the mixed effects logistic regression models comparing 12, 24, and 36 months to baseline CFA as marker for LF infection.

**Outcome**	**Treatment zone**	**Comparison**	**Adjusted odds ratio (95% CI)**	***p-*value**
CFA	Annual	Follow-up 1 vs Baseline	0.43 (0.29, 0.63)	<0.001
		Follow-up 2 vs Baseline	0.12 (0.07, 0.2)	<0.001
		Follow-up 3 vs Baseline	0.09 (0.05, 0.16)	<0.001
	Semiannual	Follow-up 1 vs Baseline	0.78 (0.6, 1.02)	0.073
		Follow-up 2 vs Baseline	0.23 (0.15, 0.33)	<0.001
		Follow-up 3 vs Baseline	0.26 (0.18, 0.37)	<0.001

**Fig. 4:**
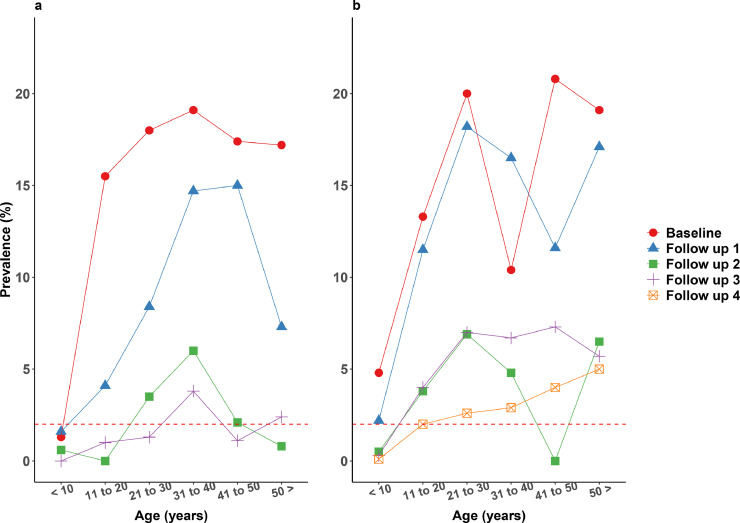
a and b: Age-prevalence profiles for circulating filarial antigenemia (CFA) by treatment zone before and after mass drug administration. The dotted red line indicates the 2% pre-TAS prevalence target.

Fig. 4 show age prevalence profiles for *W. bancrofti* infections at baseline and follow-up surveys. The patterns in the two treatment zones were similar, with higher in CFA prevalence rates in older age groups. Most age groups in the annual treatment zone had CFA prevalences below 2% in the follow-up 3 surveys, i.e. following three rounds of annual MDA. In contrast, whereas only children <10 years of age had a CFA prevalence below 2% in the semiannual treatment zone.

Table 5 shows the effect of repeated rounds of MDA on *O. volvulus* Mf prevalence estimates by treatment zone. Most onchocerciasis infections in this study area were of light or moderate intensity. MDA significantly decreased microfiladermia prevalences in both the annual (Central) and semiannual (South) treatment areas with 74.3 and 80.9% reductions from baseline in the third follow-up 3 surveys, respectively. Geometric mean Mf/mg skin and CMFL remained stable throughout in both treatment zones.

### 3.4 Impact of annual vs semiannual MDA on soil-transmitted helminth and *Schistosoma mansoni* infections

**Fig. 5:**
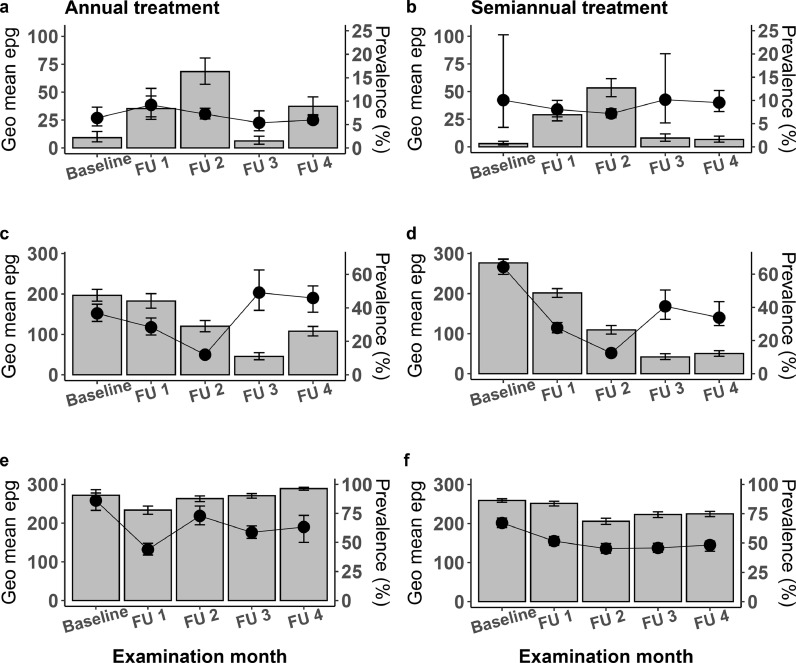
Impact of annual vs semiannual mass drug administration on helminth infections. Prevalence estimates is represented in bar graphs. Intensity (represented as geometric mean eggs per gram) are shown in line graphs. Follow-up surveys (FU); Geometric mean eggs per gram (Geo mean epg) a and b; *Ascaris lumbricoides* infection in annual and semiannual treatment zones respectively. c and d; Hookworm infection in annual and semiannual treatment zones respectively. e and f; *Schistosoma mansonia* infection in annual and semiannual treatment zones respectively. (see: Supplementary 3 for number of participants in each survey area and corresponding arithmetic mean epg).

*A. lumbricoides*, hookworm (*N. americanus)*, and *S. mansoni* infection results are summarized in Fig. 5. Data from the two semiannual treatment zones have been combined for this analysis. *Ascaris* infections were uncommon in both treatment zones at baseline, and prevalence and intensities for this infection were not dramatically affected by MDA. *S. mansoni* infections were very high in both treatment zones at baseline, and neither prevalence nor infection intensities were affected much by MDA with praziquantel. Because of the long intervals between MDA and follow-up surveys, it is likely that any short term beneficial effects of MDA on ascariasis or schistosomiasis were obscured by reinfection. The hookworm data are more encouraging, because they show that MDA reduced hookworm prevalence and intensities in both treatment zones. Although hookworm prevalences increased in the third and fourth follow-up surveys in both treatment zones, reductions in infection intensity were sustained. *Trichuris* infections were present in the study area, but results for *T. trichiura* have been excluded from this report as the data were confounded by similarly appearing *Capillaria* ova that were also present in the study area (Fischer et al., 2018).

## 4 Discussion

This study has provided extensive new data on the relative impact of three annual rounds vs five semiannual rounds of MDA with ivermectin, albendazole, and praziquantel in an area co-endemic for several major helminthic NTDs that are endemic in northwestern Liberia. Results from the follow-up 4 survey allowed us to assess whether improvements observed at the primary study endpoint were sustained by two additional rounds of routine annual MDA provided under the national NTD control program of the Liberia Ministry of Health.

The study results show that semiannual MDA was no more effective than annual MDA for clearing *W. bancrofti* CFA in this setting. The Liberia Ministry of Health/African Programme for Onchocerciasis Control had conducted MDA using ivermectin for onchocerciasis control a few months prior to our baseline survey, and we believe that this was responsible for the low baseline *W. bancrofti* Mf rates recorded during our survey. Thus it was difficult to assess the impacts of MDA on Mf prevalence. Results from this study are consistent with results from parallel studies that were conducted in Maryland County in southeastern Liberia (Eneanya et al., 2021), in Côte d'Ivoire (unpublished), in Papua New Guinea (unpublished), and in Flores Island Indonesia (*Brugia timor*i) (Supali et al., 2019). The WHO based their recommendation for annual MDA for LF elimination was based in part on preliminary results from these studies (WHO 2017).

A mathematical modeling study predicted that more frequent MDA would significantly reduce the time required for LF elimination and reduce overall program costs in areas endemic for *W. bancrofti* .^9^ Although this may be true in some settings, data from the present study and other studies cited above are not consistent with the model predictions. For areas with low to moderate baseline prevalence, we believe that annual MDA is sufficient for LF elimination if high rates of treatment adherence can be achieved (Campillo et al., 2021).

Prior to our study, there was sparse recent data published on LF infection markers in Liberia. Kuhlow and Zielke (1976) in their 1976 study reported an Mf prevalence of 11.4% in the savanna areas in the northwestern region of the country. Consistent with this, pilot surveys conducted in 7 MDA-naïve villages in this region by our field team for study site selection in 2011 shows that *W. bancrofti* Mf prevalence ranged between 7.9 and 16.0% (10.0% average), and CFA prevalence ranged from 10.0 and 28.8% (21.5% average) [see Supplementary 2]. The Mf prevalence in the baseline survey was only 1.7%, (as mentioned above), this low value was probably due to ivermectin MDA that was provided by the Ministry of Health in the study area between the time of our pilot surveys and the baseline survey. High baseline CFA prevalences in the Central and North treatment zones in this study are consistent with that explanation, because ivermectin is efficient for clearing Mf without clearing CFA. Other studies have shown that Mf prevalence declines much faster than antigenemia following MDA with ivermectin and albendazole (Ismail et al., 1998), although the relationship between these parameters after treatment is non-linear and complex (Cano et al., 2015; Irvine et al., 2016).

Age-prevalence curves illustrate a weakness of TAS surveys as stopping criteria for LF elimination programs. CFA prevalence in young children was low in this study area with moderate LF endemicity prior to initiation of MDA with ivermectin plus albendazole. This finding should make it difficult for authorities to have much confidence in TAS as a stopping criteria for MDA in areas like Lofa County. Similarly our finding that community CFA prevalences remained well above 2% long after Mf prevalences had been reduced to less than 1% suggests that WHO's 2% pre-TAS target for CFA is much more stringent than the older pre-TAS Mf prevalence target of 1%. We recently reported a similar finding from a study conducted in a different area in Liberia that had a higher baseline LF prevalence than that in Lofa County (Eneanya et al., 2021). Thus, reliance on a 2% target for pre-TAS surveys will often require additional rounds of MDA beyond the number required to reduce Mf prevalence below 1%.

It is interesting that the remarkable decreases in LF prevalence were achieved despite unavoidable interruptions in our study related to the 2014 Ebola outbreak (Swanson et al., 2018) which delayed MDA distribution and the SARS-CoV-2 pandemic which delayed the timing of our final follow-up survey by a full year (Wu et al., 2020). The continued declines in LF prevalence recorded in this study are in keeping with predictions from recent mathematical modeling work that examined the impact of missed or delayed MDA rounds on the resurgence of NTD infections (Toor et al., 2021). In that study, Toor et al. predicted that LF resurgence would be unlikely following one missed MDA round. Progress towards elimination was maintained if treatment coverage of >65% was achieved when MDA resumed. Higher resurgence rates were predicted for infections with helminth species such as those that cause STH infections because of their shorter life spans(Dunn et al., 2019; Fulford et al., 1995). That is because resurgence rates are approximately proportional to 1/L, where L is the lifespan of the worm in the human host. Slower resurgence rates are expected for infections with *W. bancrofti* or *O. volvulus* with worm lifespans in the range of 5–15 years (Kwarteng and Ahuno, 2017; Anderson and May, 1991).

Our results showed that LF infections were more common at baseline in older people. This is probably because adults have had more time to become infected, as it requires prolonged exposure to mosquitoes for infections to be initiated. At follow-up 1, persons who reported prior compliance with MDA had lower odds of LF infection. Previous studies have reported that treatment compliance was an important factor for successful control programs and key in achieving elimination targets (Campillo et al., 2021; Krentel et al., 2013), and filarial infection prevalences also responded well to community MDA in the present study. The finding that males had higher LF prevalences that females is consistent with previous studies in Liberia (Eneanya et al., 2021), and elsewhere (Bundy, 1988). A review on gender and filariasis found that lower rates of infection may be attributed to less exposure of adult females to infective vectors (Brabin, 1990) (Table 5).

**Table 5:** *O. volvulus* microfiladermia prevalence estimates and infection intensities following MDA by treatment zone.

	**Baseline**	**Follow-up 1**	**Follow-up 2**	**Follow-up 3**	**Follow-up 4**	***p* value(Baseline – follow-up 3)**
**Annual MDA** n = 3510	n = 1008	n = 525	n = 494	n = 706	n = 804	
Prevalence (95% CI)	14.4 (12.3, 16.7)	9.1 (6.8, 11.9)	5.5 (3.6, 7.9)	3.7 (2.5, 5.6)	2.9 (1.9, 4.4)	0.012†
Geometric Mf/mg skin (95% CI)	2.5 (2.1, 3.2)	2.5 (1.7, 3.8)	1.7 (1.1, 2.5)	1.3 (1.0, 1.7)	1.5 (1.2, 1.8)	0.91
Community Mf load (CMFL)	1.1 (1.0, 1.3)	1.1 (0.9, 1.4)	0.9 (0.7, 1.2)	0.8 (0.7, 1.0)	1.0 (0.8, 1.3)	0.995
**Class of intensity (%)**						
Light (<10 Mf/mg skin)	84.1 (77.2, 89.7)	79.2 (65.0, 89.5)	92.6 (75.7, 99.0)	100 (51.7, 99.7)	97.8 (55.2, 98.2)	0.758
Moderate (11-30 Mf/mg skin)	10.3 (5.9, 16.5)	18.8 (8.9, 32.6)	7.4 (0.9, 24.2)	0 (0.3, 48.2)	2.2 (1.1, 8.9)	0.113
Heavy (>30 Mf/mg skin)	5.6 (2.4, 10.6)	2.0 (0.05, 11.1)	0 (0, 12.8)	0 (0, 33.6)	0 (0, 21.9)	0.012
						
**Semiannual MDA** n = 3310	n = 1142	n = 367	n = 322	n = 198	n = 1281	
Prevalence (95% CI)	23.6 (21.2, 26.2)	27.8 (23.2, 32.7)	18.6 (14.5, 23.3)	4.5 (2.1, 8.5)	4.4 (3.4, 5.7)	< 0.001†
Geometric Mf/mg skin (95% CI)	2.9 (2.6, 3.3)	3.2 (2.4, 4.2)	1.4 (1.1, 1.8)	1.7 (0.7, 4.1)	2.1 (1.6, 2.7)	0.797
Community Mf load (CMFL)	1.2 (1.1, 1.3)	1.3 (1.1, 1.4)	0.8 (0.7, 0.9)	0.9 (0.5, 1.5)	1.1 (0.8, 1.6)	0.984
**Class of intensity (%)**						
Light (<10 Mf/mg skin)	83.4 (79, 87.2)	78.4 (69.2, 86.0)	95.0 (86.1, 99.0)	88.9 (51.7, 99.7)	96.2 (88.3, 98.2)	0.631
Moderate (11-30 Mf/mg skin)	13.6 (10.2, 17.9)	14.7 (8.5, 23.1)	3.3 (0.4, 11.5)	11.1 (0.2, 48.2)	3.8 (2.2, 4.3)	0.059
Heavy (>30 Mf/mg skin)	3.0 (1.4, 5.4)	6.9 (2.8, 13.6)	1.7 (0.04, 8.9)	0 (0, 33.6)	0 (0, 18.2)	0.03

Villages in the Center treatment zone received annual MDA while villages in the South received semiannual MDA.

Numbers shown are percentages (95% confidence intervals).

† *p* value compares baseline to follow-up 3.

Reported use of bed nets and window screens was not linked to reduced baseline odds of LF infection in this study. This is in contrast with studies elsewhere which reported protective effects of bed nets and other vector control measures (Chesnais et al., 2014; Eigege et al., 2013). Bed net data reported in our study may have been inaccurate, because the survey only asked whether participants had used a bed net on the night before the survey. This may have obscured a true protective effect of bed net use.

Various socio-economic factors have been previously linked to LF infection. Studies have reported that individuals from poorer households were more predisposed to infection (Upadhyayula et al., 2012; Gyapong et al., 1996). From our study, household water source was taken as proxy for water, sanitation, and hygiene. We found that households who had access to water from a shared public pump (rather than from rivers and streams) had lower odds of infection, as has been reported elsewhere (Gyapong et al., 1996).

Treatment with ivermectin plus albendazole is known to have little lasting effect on adult *O. volvulus* worms (Keiser et al., 2002; Churcher et al., 2009), and many years of ivermectin are required to eliminate onchocerciasis in meso- and hyperendemic settings. Although baseline skin Mf prevalences in our study sites were in the hypoendemic range (skin Mf prevalence <30%), pilot surveys for site identification conducted in this area 6 months prior to baseline recorded skin Mf prevalences as high as 63% (see Supplementary 2). Our results show that annual MDA with ivermectin plus albendazole was as effective as semiannual MDA in this setting, and that annual MDA provided by the Liberia Ministry of Health was sufficient to sustain improvements that occurred during our study. These results are better than those observed in a parallel study that was performed in southeastern Liberia (Eneanya et al., 2021) where microfiladermia was only modestly affected by MDA. Although CMFL was low at baseline, ivermectin plus albendazole was not effective for reducing this further. A few heavily infected persons that did not comply with MDA can contribute to stable CMFL values within a community (Remme et al., 1986; Remme et al., 1990). While these numbers are encouraging, it is clear that MDA with ivermectin cannot eliminate onchocerciasis in this time frame. More rounds of ivermectin MDA will potentially result in the sterilization of female adult worms. Also, older worms are known to have reduced capacity for Mf production, even in the absence of treatment (Duke, 2005).

Soil-transmitted helminth infections are widespread in Liberia, but recent data are limited. A recent publication reported moderately high prevalences for Ascaris, hookworm, and Trichuris infections in Eastern Liberia prior to MDA. An early study of the distribution of hookworm in Liberia found a prevalence of 94% in northern Lofa County in 1972 (Hsieh et al., 1972). The present study found that hookworm was the dominant STH infection in this area, with a baseline prevalence of 66% in in 2012. Infection prevalences and egg counts decreased similarly following annual or semiannual MDA with ivermectin plus albendazole. However, the rebound in hookworm prevalence documented in the last two follow-up surveys reinforces the point MDA alone cannot eliminate hookworm infections in settings with high baseline prevalences (Dunn et al., 2019). While MDA is an effective tool for controlling STH in the short term, elimination will require an integrated approach of MDA coupled with improved sanitation and hygiene.

Kato-Katz is the most commonly used diagnostic technique for detecting and quantifying STH infections in field surveys. However this technique is less sensitive quantitative polymerase chain reaction (qPCR) assays in areas with light infections (Dunn et al., 2020). When we used qPCR to test stool samples from this study for quality control, we found that many samples that had been classified as positive for *T. trichiura* by Kato-Katz were negative by qPCR. Further investigation showed that this was due to misidentification of *Capillaria* eggs as being *T. trichiura* which have a similar size and appearance to *Capillaria* by Kato-Katz (Fischer et al., 2018). This problem confounded results for *T. trichiura* in this study, and they were not reported here for that reason.

Many parts of Liberia including our study sites in Lofa County are highly endemic for *Schistosoma mansonia*. The prevalence and intensity of *S. mansoni* infections remained high in our study area despite annual or semiannual MDA with praziquantel. Although we cannot exclude parasite resistance to praziquantel, the ineffectiveness of MDA is presumably due to reinfection (Farrell et al., 2017; Shuford et al., 2016). Based on this, we conclude that MDA with praziquantel is not sufficient to eliminate or control *S. mansoni* infection in this hyperendemic setting.

In conclusion, our study has provided extensive new data on the impact of MDA with albendazole, ivermectin, and praziquantel on helminthic NTDs. MDA had the greatest impact on filariasis, a lesser impact on onchocerciasis and STH, and little impact on schistosomiasis. Integrated approaches will be needed to control or eliminate STH and schistosomiasis in areas like Lofa County. Annual MDA was as effective as semiannual MDA for the nematode parasites targeted in our study. We therefore suggest that NTD elimination programs should focus on delivering high quality annual MDA with high coverage and compliance and not try to stretch limited resources to deliver semiannual MDA.

## Financial support

This project was funded by grant GH5342 from the Bill & Melinda Gates Foundation to the DOLF Project (https://dolfproject.wustl.edu/). The funders had no role in the study design, data collection and analysis, decision to publish, or preparation of the manuscript. The findings and conclusions contained within are those of the authors and do not necessarily reflect positions or policies of the Bill & Melinda Gates Foundation.

## Disclosures

The filarial antigen test used in this study uses reagents licensed from Barnes-Jewish Hospital, an affiliation of Gary. J. Weil. All royalties from sales of these tests go to the Foundation for Barnes-Jewish Hospital (https://www.foundationbarnesjewish.org/), a registered not-for-profit organization.

## CRediT authorship contribution statement

**Obiora A. Eneanya:** Formal analysis, Software, Visualization, Writing – original draft, Writing – review & editing. **Lincoln Gankpala:** Investigation, Project administration. **Charles W. Goss:** Formal analysis, Software, Writing – review & editing. **Aaron T. Momolu:** Investigation. **Enoch S. Nyan:** Investigation. **Emmanuel B. Gray:** Investigation. **Kerstin Fischer:** Investigation. **Kurt Curtis:** Investigation. **Fatorma K. Bolay:** Project administration. **Gary J. Weil:** Conceptualization, Funding acquisition, Project administration, Writing – review & editing. **Peter U. Fischer:** Conceptualization, Funding acquisition, Project administration, Writing – review & editing.

## Declaration of Competing Interest

The authors declare that they have no known competing financial interests or personal relationships that could have appeared to influence the work reported in this paper.

## Appendix Supplementary materials

Image, application 1

Image, application 2

Image, application 3
