# Supplementary material for: Community-based trial assessing the impact of annual versus semiannual mass drug administration with ivermectin plus albendazole and praziquantel on helminth infections in northwestern Liberia
Source: Acta Trop. 2022 Jul;231:106437. doi: 10.1016/j.actatropica.2022.106437 (PMC9168441; doi:10.1016/j.actatropica.2022.106437)
Supplement: Supplementary file 1 [file mmc1.docx]

**Supplementary 1**: Mass drug administration (MDA) compliance rates stratified by North, Center, and South treatment zones

| **Variable** | **North treatment zone** | **Center treatment zone** | **South treatment zone** |
| --- | --- | --- | --- |
| **Surveyed MDA compliance^1^** |  |  |  |
| Baseline | NA | NA | NA |
| Follow up 1 | 83.2 | 72.5 | 70.3 |
| Follow up 2 | 84.8 | 74.0 | 76.8 |
| Follow up 3 | 69.7 | 64.2 | 64.4 |
| Follow up 4 | 53.8 | 55.6 | 50.1 |

^1^ MDA-compliant participants were those who reported having swallowing albendazole and ivermectin in the previous round of MDA at the time of the next round.
