# Supplementary material for: Community-based trial assessing the impact of annual versus semiannual mass drug administration with ivermectin plus albendazole and praziquantel on helminth infections in northwestern Liberia
Source: Acta Trop. 2022 Jul;231:106437. doi: 10.1016/j.actatropica.2022.106437 (PMC9168441; doi:10.1016/j.actatropica.2022.106437)
Supplement: Supplementary file 2 [file mmc2.docx]

**Supplementary 2**: Results of pilot surveys for lymphatic filariasis (antigenemia and microfilaremia) and *Onchocerca volvulus* (skin microfilaria and nodule palpation) prevalences in villages in Lofa County prior to the baseline surveys.

| **Village within Lofa County** | **Number of participants** | **Lymphatic filariasis antigenemia**  **% (95% CI)** | **Lymphatic filariasis microfilaremia**  **% (95% CI)** | ***Onchocerca volvulus* nodule**  **% (95% CI)** | ***Onchocerca volvulus* microfiladermia %** |
| --- | --- | --- | --- | --- | --- |
| **Wabengu** | 63 | 20.0 (10.1-29.9) | 7.9 (1.2-14.6) | 31.7 (19.6-42.4) | 63 |
| **Sakawa** | 52 | 19.2 (8.3-29.7) | 9.6 (1.7-17.6) | 17.3 (6.8-27.2) | 35 |
| **Lepalo** | 52 | 28.8(15.8-40.2) | 7.7 (0.5-15.0) | 9.6 (1.6-17.6) | 19 |
| **Kenema** | 50 | 28.0 (15.6-40.4) | 16.0 (5.8-26.1) | 6.0 (0-12.6) | 12 |
| **Kilima** | 50 | 22.0 (10.5-33.5) | 8.0 (0.5-15.5) | 6.0 (0-12.6) | 12 |
| **Medikorma** | 50 | 22.0 (10.5-33.5) | 10.0 (1.7-18.3) | 0 | 0 |
| **Foya Dundu** | 50 | 10.0 (1.7-18.3) | 10.0 (1.7-18.3) | 14 (4.4-23.6) | 28 |
| **Total** | 367 | 21.5 (9.7-32.3) | 10.0 (1.7-18.3) | 12.8 (3.0-21.0) | 25 |

The pilot field surveys conducted in Lofa County were performed to identify suitable sites for the community mass drug administration studies. These villages had not received mass drug administration with either ivermectin or albendazole when the pilot surveys were performed.
